# This meat or that alternative? How masculinity stress influences food choice when goals are conflicted

**DOI:** 10.3389/fnut.2023.1111681

**Published:** 2023-03-07

**Authors:** R. Bret Leary, Rhiannon MacDonnell Mesler, William J. Montford, Jennifer Chernishenko

**Affiliations:** ^1^College of Business, University of Nevada, Reno, NV, United States; ^2^Dhillon School of Business, University of Lethbridge, Lethbridge, AB, Canada; ^3^Coggin College of Business, University of North Florida, Jacksonville, FL, United States; ^4^Kent Business School, University of Kent, Canterbury, United Kingdom

**Keywords:** masculinity, masculinity stress, red meat, meat alternatives, PBMAs, goal conflict, impression management

## Abstract

**Introduction:**

This research integrates literature on masculinity stress—the distress experienced as the result of a perceived discrepancy with male gender norms—with research on goal conflict to examine preferences for plant-based meat alternatives (PBMAs). Men experiencing masculinity stress are likely to hold salient a goal of being masculine, which should lead to less preference for PBMAs. However, many of these men simultaneously hold competing goals, such as making ethical food choices, which remain inhibited in favor of the focal masculinity goal. We argue that once men experiencing masculinity stress highlight their masculinity through the selection of a manly product, they satisfy that higher-order goal and are then free to pursue previously inhibited goals, such as making an ethical choice through the selection of PBMAs.

**Methods:**

We present the results of three studies supporting these expectations. Study 1 tests the link between masculinity stress and meat (alternative) consumption using consumer search behavior collected from Google Trends, showing that masculinity stress is positively (negatively) correlated with searches for red meat (PBMAs). Study 2 shows that men experiencing masculinity stress are more inclined to choose PBMAs, provided they are presented within a masculine product context. Study 3 presents a parallel mediation model, showing that ethical considerations (as opposed to masculine goals) shape the choice of PBMA preference.

**Results and discussion:**

We conclude with a discussion of theoretical implications for the impression management strategies utilized by men experiencing masculinity stress and practical implications for the growing PBMA industry.

## 1. Introduction

### 1.1. Plant-based meat alternatives

Increasing concerns about the health and environmental impacts of red meat consumption (1–3) have led to a recent surge in popularity of plant-based meat alternatives (PBMAs),^1^ allowing consumers the opportunity to eat healthier while leaving a smaller ecological footprint. While many types of plant-based alternative proteins are available to consumers, including (but not limited to) protein from legumes/pulses, algae, and soy, we limit our focus on plant-based meat alternatives to modern forms that are intended to replicate the taste and texture of real meat. Doing so allows for a comparison of similar products (i.e., a burger) that contain different attributes (i.e., real meat patty vs. meat alternative).

Sales of PBMAs reached almost $30 billion in 2020 and are projected to exceed $160 billion by 2030—what amounts to an estimated 10% of the global meat market (4). The growing prevalence of meat alternatives offered by restaurants supports such projections, as the number of US restaurants offering them grew 27% from 2017 to 2019, while restaurant sales of meat alternatives grew 268% during the same time frame (5). Indeed, companies like Impossible Foods and Beyond Meat are leading the charge for this industry, gaining recognition and widespread distribution through restaurants like Burger King, Hardee's, Starbucks, Qdoba, Cheesecake Factory, Little Caesar's, and TGI Friday's, just to name a few. This marketing and promotion strategy has been successful, as over 70% of restaurant patrons claim to be aware of PBMA offerings in restaurants, with 54% of these individuals having tried them—a majority of whom identify as meat eaters (6).

The rise in demand and acceptance of PBMAs in recent years lies primarily in the use of innovative food technology to close the taste gap with real meat. For example, Impossible Foods pioneered the use of heme, a compound derived from soybeans, to simulate the appearance, flavor, aroma, and texture of red meat (7). The authenticity of heme creates almost imperceptible differences from real meat, allowing for a nearly seamless transition to meat alternatives with very limited trade-offs in taste. Likewise, Beyond Meat adds beet juice extract to its PBMA offerings to provide the appearance of a “bleeding” beef burger (8). Though questions arise as to the health benefits of highly processed meat alternatives (9), they are positioned by manufacturers as a healthful alternative to red meat (10, 11) and consumers perceive them as such (12, 13), making it likely that consumers use them to try and achieve their health goals.

Meat alternatives also have a lower carbon footprint than red meat (14) and require substantively lower resource intensity (e.g., water) to produce (15). For example, Beyond Meat claims to use 46% less energy, 99% less water, and 93% less land, while producing 90% less greenhouse gases (GHG) than beef production (16); Impossible Foods likewise states their products use 87% less water, 96% less land, and produce 89% less GHG (17). As such, meat alternatives offer an environmentally friendly option to red meat. Combined with the fact that they pose fewer ethical concerns for animal welfare (18), it is evident that PBMAs provide individuals with the opportunity to potentially achieve their health, ethical, and environmental consumption goals, thus serving as a possible first step in transitioning away from a meat-based diet. Taken together, it seems likely that PBMAs will remain popular for the foreseeable future, which is important given the general consensus among health experts that alternatives to red meat can help improve one's health (19).

Despite the promoted benefits of PBMAs, questions remain about the individual-level factors that facilitate or inhibit acceptance of these products. For example, Graça, Oliveira, and Calheiros (20) found that affective connection to meat (ranging from disgust to hedonic feelings) and intensity of attachment to meat influenced willingness to reduce its consumption. Likewise, culture also influences meat preferences. Bryant et al. (21) show that Indian and Chinese consumers were more likely than U.S. consumers to purchase plant-based or clean (i.e., lab) meat. These same authors also show that food neophobia (i.e., the avoidance of new foods) and meat attachment impact meat preferences, with Indian consumers tending to be more food neophobic and having less attachment to meat than those in China and the U.S. Personality traits and political orientation also impact meat consumption, with Pfeiler and Egloff (22) finding that the traits of openness and agreeableness are negatively related to the consumption of meat, while conservatism has a positive relationship.

One of the most studied individual differences relating to meat consumption is gender. Generally, the literature suggests that males prefer (and eat) more meat than females, while preferring plant-based proteins less than females [e.g., (2, 20, 23)]. This preference has evolutionary underpinnings, such that men have the need to position themselves as an attractive mate, heightening one's desire to signal their masculinity to potential partners through meat consumption (24–26). However, while research explains this phenomenon by broadly focusing on traditional gender norms and societal expectations, recent research suggests there is more nuance in *how* these norms and expectations influence meat consumption. For example, the association between red meat consumption and masculinity (27–31) implies that men should be less likely to adopt a diet consisting of meat alternatives, wherein it could potentially threaten their masculine status as non-meat diets are linked with lower perceived masculinity (32). This association is perpetuated through disinformation in popular media suggesting, for instance, that meat alternatives like Impossible Burgers contain more estrogen than what is in gender transformation treatments; in short, eating such products will “give you breasts” and “make you female” (33, 34). Further, Hinrichs et al. (35) found that males reacted more defensively than females against plant-based eating, noting that males were more likely to perceive plant-based eating as a threat to their wellbeing, engage in psychological reactance (e.g., be more pro-meat after viewing a pro-*plant-based* message), and practice moral disengagement (e.g., bolster specific values by deactivating certain morals to reduce cognitive dissonance and negative feelings like guilt).

Thus, existing research suggests that men who want to appear masculine (or who are worried that they are not masculine enough) might be less inclined to prefer PBMAs. At the same time, however, there has been a dramatic increase in the number of men who report having personal health and environmental goals [e.g., (36)]—goals which could potentially be achieved by reducing red meat consumption (37) or, alternatively, increasing their consumption of meat alternatives (38). Therefore, we ask, how might men who are concerned about signaling their masculinity be motivated to consume meat alternatives, even though doing so could potentially help them achieve other food-related goals?

Building from this, we argue that many male consumers simultaneously hold incongruent and competing food-related goals at any given time. For example, they may have a goal of eating something tasty or something healthy, which are intuitively different in the minds of individuals (39). Likewise, they may have the self-concept-related goal of living up to socialized gender norms by making “manly” choices, which can be incongruent to the goals of eating healthy (40) or making ethical and environmentally friendly choices (41). When faced with such conflicting goals, these men must determine which one to act upon at any given time.

The current research explores this dilemma by examining how men faced with these conflicting goals balance them and prioritize one over the other. To do so, we integrate research on *masculinity stress* (42, 43) with research on *goal conflict* (44–46). Defined as the “on-going distress experienced as the result of a perceived discrepancy with male gender norms” [(31), p. 2], masculinity stress represents how central the masculine goal is to a man by reflecting the degree to which a man believes he is (in)sufficiently masculine (42). The goal conflict literature, which provides insight into how individuals balance competing goals and determine which ones are active at any given point and thus acted upon, can be used to explain how men experiencing masculinity stress reconcile seemingly incompatible goals. By combining these two literature streams, we provide insight into attitudes toward the emerging meat alternative market and some of the factors influencing its acceptance among men who are simultaneously balancing the competing goals of signaling their masculinity while also eating ethically.

### 1.2. Masculinity, masculinity stress, and red meat consumption

Masculinity is a social identity manifest through a set of stereotypical traits and behaviors (47). While there are many highly visible outlets through which men choose to display their masculinity, including gun support and ownership (48), gambling (49), alcohol/substance abuse (43, 50), and vehicle choice (51), one common way in which men do so is through their food choice. Red meat, in particular, is associated with masculinity (28, 30) and masculine qualities such as virility and sexual strength (52), mating desirability (24), and status (25). Indeed, red meat consumption is viewed as implicitly and explicitly masculine (53) by both men and women (29, 32). Conversely, a vegetarian and/or vegan diet can signal that one is less masculine than others (54). Perhaps unsurprisingly, then, men consume significantly more meat than women and are less likely to become vegetarian (55), making the acceptance of meat alternatives in many ways a gendered issue.

Despite this seemingly ubiquitous connection, however, not all men uniformly experience the desire or need to demonstrate their masculinity through stereotypical masculine behavior like meat consumption. Indeed, whereas research (56) shows that certain men, particularly those adopting more traditional attitudes about masculinity, are more likely to feel compelled to engage in masculinity-signaling behavior, males espousing “new masculinity” ideals, as reflected by more progressive beliefs about gender roles (57), display a weaker attachment to red meat and, consequently, are more likely to reduce their meat consumption. Stated differently, within-gender differences exist in the relationship between masculinity and meat consumption, making it important to understand what drives men to consume less meat and/or more meat alternatives.

Recognizing these within-gender differences, we argue that the “traditional” masculine goal is not equally salient among all men, and that the desire to display one's masculinity through food choice will be higher among men high on masculinity stress. That is, the goal of engaging in “manly” behavior should be heightened for men higher (vs. lower) in masculinity stress, as those who believe they are not living up to expected masculine ideals will ostensibly be more likely to display their masculinity through their behavior, particularly by consuming more meat. In line with this theorizing, recent research by Mesler et al. (31) finds that masculinity stress is associated with preference for consuming red meat products. Specifically, those experiencing heightened distress as a result of a perceived discrepancy with masculine norms were more likely to prefer meat, based on the belief that doing so would augment their perceived deficient masculinity. However, these authors do not explore the acceptance of meat *alternatives* among men experiencing masculinity stress which, given recent research [e.g., (35, 55)], should be negatively associated with one another. Accordingly, we predict that men who are high on masculinity stress, and thus deem themselves to not be living up to desired masculine standards, will be more likely to seek out meat products and, concurrently, less likely to pursue meat alternatives. Stated formally:

H1: Masculinity stress will be positively associated with the pursuit of red meat and negatively associated with meat alternatives.

However, even men high on masculinity stress are likely to have more than one goal in making food choices; a desire to be seen as manly doesn't preclude a man from also desiring to eat healthfully or ethically. Indeed, recent research [e.g., (36)] shows that men—even those focused on traditional indicators of masculinity like physique, sexual prowess, and career dominance—are more active in pursuing goals related to health and the environment than previously expected. As such, we believe it worthwhile to ask, what about men who have the goal of displaying their manliness through their food choice but also hold a goal of eating healthy and/or making environmentally friendly food choices—goals which are incongruent to, and likely in competition with, their masculinity goals? To address this question, we turn to the literature on goal conflict.

### 1.3. Goal conflict

Goal conflict exists when an individual holds two or more goals which often cannot be satisfied through a single action, such that the pursuit of one goal harms the pursuit of another (58). Perhaps unsurprisingly, then, goal conflict significantly impacts goal achievement, as pursuing multiple goals simultaneously makes attaining either one less likely—a dilemma that increases with the degree of conflict between the goals (59, 60). Thus, men who hold both a goal of being masculine (as represented by a heightened experience of masculinity stress) and making a healthy or environmentally conscious food choice will likely struggle to achieve both simultaneously.

Laran and Janiszewski (46) propose a passive goal guidance model of goal conflict in such situations, which argues that the management of goal conflict results from both activation and inhibition of competing goals. In this model, lower-order goals are inhibited while superordinate goals are activated for pursuit, which remain active until completion and the individual is free to pursue the heretofore inhibited goals. Importantly, this process occurs at an unconscious level, such that individuals do not purposefully or knowingly switch between active goals. Laran and Janiszewski (46) demonstrate that behavioral consistency in pursuit of a goal is the result of a focal goal remaining active (e.g., because it has not yet been achieved), while competing goals remain inhibited, and behavioral *in*consistency is the result of a previously activated goal being achieved, and in turn, a previously inhibited (or inactive) goal becoming activated. For example, when it comes to meat and meat alternatives, men who are higher on masculinity stress should be more likely to both hold and have salient the goal of making “manly” food choices, while other goals that they hold around their food consumption (e.g., healthy, ethical, etc.) may remain inhibited.

However, in order to compel men high on masculinity stress to choose meat alternatives, which we argue herein is inconsistent with the goal of being “manly,” the passive goal guidance model (46) suggests that making a masculine food choice (such as choosing a burger) should achieve the “manly” goal and thus allow previously inhibited goals to become activated (e.g., health or ethical goals). In turn, these men might be encouraged to then choose an attribute *within* those products that satisfy their newly activated goals, such as selecting a PBMA, even if it is considered potentially less masculine. Stated differently, the selection of the masculine *product* (the burger) should serve to signal one's masculinity, which then frees one to opt for a potentially less manly *attribute* like meat alternatives. In this way, we distinguish between the effects of masculinity stress at the product vs. the attribute level and examine whether intra-product differences impact food choice among those high on masculinity stress.

Men who are low on masculinity stress, in contrast, should hold the “manly” goal less strongly, and in turn be less subject to this effect, given that this goal is less salient in their decision-making process. Thus, we predict that once men who are experiencing masculinity stress have the opportunity to highlight their masculinity through the selection of a “manly” product, they will have achieved that goal and have the liberty to pursue alternative, previously inhibited goals. It follows, then, that if men are able to satisfy their masculinity goals through the selection of a masculine food product, then other goals will be free to drive the selection of meat alternatives. Stated formally, we predict:

H2: Men high on masculinity stress will be more likely to choose a meat alternative than those low on masculinity stress, provided it is presented within a masculine product.

H3: The satisfaction of the masculinity goal through the selection of a manly food product allows other goals (previously inhibited) to drive the selection of meat alternatives.

## 2. Overview of studies

We present the results of three studies testing the hypotheses. Study 1 tests the link between masculinity stress and meat (alternative) consumption using real consumer search behavior collected from Google Trends (H1). Leveraging the intent of modern meat alternatives to replicate real meat, Study 2 has participants choose within a masculine product context (i.e., a burger), between one with masculine (red meat) and one with non-masculine (non-meat) attributes. This allows us to determine whether men who are high in masculine stress will be more likely to choose meat alternatives, provided they are able to signal their masculinity through the selection of a masculine product (H2). Finally, Study 3 tests the expectation that the selection of a masculine product allows other goals to shape the choice of meat alternatives by running a parallel mediation model with masculinity-seeking and ethical consumption goals as mediators between masculinity stress and meat alternative choice (H3).

### 2.1. Study 1: Linking masculinity stress to meat consumption through Google search behavior

#### 2.1.1. Method

As preliminary evidence of the link between masculinity stress and meat consumption, we study real consumer search behavior. Data were collected from Google Trends (https://trends.google.com) for a 1-year time period (June 2018–19) on search behavior in all metro areas across the United States (*N* = 210). We focused first on a cluster of search terms (*n* = 9; α = 0.781) that were chosen, based on previous research (61), to represent factors underlying masculinity stress, including “erectile dysfunction,” “penis enlargement,” and “how to get girls.” Next, we chose three individual search terms representing red meat (“burger,” “BBQ,” and “steak”) and two meat alternatives (“Beyond Meat” and “Impossible Burger”) that emerged from a detailed pretest. Finally, we selected general terms to represent base-rate search behavior for use as a covariate, including “weather,” “YouTube,” “Facebook,” and “Amazon” (*n* = 4; α = 0.734).

#### 2.1.2. Results and discussion

Google Trends data are presented as the relative popularity of a topic in a certain region during a certain time, ranging from 0 (no popularity/not enough data for a trend) to 100 (the most popular point for the topic). The level of popularity for each topic is represented as the mean in Table 1. On average, the control variables used for the base-rate search behavior (M_Amazon_ = 68.10, M_Facebook_ = 56.15, M_Weather_ = 67.97, M_YouTube_ = 52.72) were more popular than the meat alternative terms (M_BeyondMeat_=30.41, M_ImpossibleBurger_=32.17). However, the masculine terms for specific meats (M_Steak_ = 52.62) and burger (M_Burger_ = 63.41) were just as popular as the base-rate search terms, except for BBQ (M_BBQ_ = 42.82) which was between the base-rate/specific meat search terms and the meat alternative search terms.

**Table 1:** Study 1 correlations and descriptives.

**Control variables**		**Mean**	**SD**	**Alpha**		**BBQ**	**Burger**	**Steak**	**Beyond meat**	**IMP Burger**	**Manly search terms**	**Masculinity stress**
Search behavior base rate (4 items; alpha = 0.734)	BBQ	46.69	16.97	–	Correlation	1.000	0.137	0.102	−0.073	−0.066	0.176	0.254
					Sig (2-tailed)		0.199	0.340	0.498	0.537	0.099	0.016
					df	0	87	87	87	87	87	87
	Burger	65.28	9.82	–	Correlation	0.137	1.000	0.013	0.080	−0.028	0.198	0.231
					Sig (2-tailed)	0.199		0.905	0.455	0.797	0.063	0.029
					df	87	0	87	87	87	87	87
	Steak	53.47	9.82	–	Correlation	0.102	0.013	1.000	−0.292	−0.005	0.208	0.153
					Sig (2-tailed)	0.340	0.905		0.005	0.966	0.050	0.153
					df	87	87	0	87	87	87	87
	Beyond meat	32.27	16.19	–	Correlation	−0.073	0.080	−0.292	1.000	0.612	−0.487	−0.370
					Sig (2-tailed)	0.498	0.455	0.005		<0.001	<0.001	<0.001
					df	87	87	87	0	87	87	87
	IMP burger	33.56	16.03	–	Correlation	−0.066	−0.028	−0.005	0.612	1.000	−0.486	−0.310
					Sig (2-tailed)	0.537	0.797	0.966	<0.001		<0.001	0.003
					df	87	87	87	87	0	87	87
	Manly search terms	50.05	6.48	0.774	Correlation	0.176	0.198	0.208	−0.487	−0.486	1.000	0.351
					Sig (2-tailed)	0.099	0.063	0.050	<0.001	<0.001		<0.001
					df	87	87	87	87	87	0	87
	Masculinity stress	54.87	6.35	0.781	Correlation	0.254	0.231	0.153	−0.370	−0.310	0.351	1.000
					Sig (2-tailed)	0.016	0.029	0.153	<0.001	0.003	<0.001	
					df	87	87	87	87	87	87	0

Descriptives and zero-order (Table 1) controlling for base-rate search behavior are presented. Because base-rate search behavior was significantly correlated with many search terms, partial correlations were interpreted. Interestingly, greater search for the terms that may reflect masculinity stress (i.e., “erectile dysfunction,” “penis enlargement,” and “how to get girls”) was significantly positively correlated with searches for “burger” (*r* = *0.2*3, *p* < 0.05) and “BBQ” (*r* = 0.25, *p* < 0.05) and positively, but non-significantly, correlated with searches for “steak” (*r* = 0.15, *p* = 0.15), as expected. Conversely, greater search for the terms that may reflect masculinity stress was significantly negatively correlated with searches for “Beyond Meat” (*r* = −0.37, *p* < 0.01) and “Impossible Burger” (*r* = −0.31, *p* < 0.05). Thus, preliminary evidence exists of a relationship between masculinity stress and red meat consumption (vs. meat alternatives).

Hierarchical multiple regression was next performed to examine the effects of masculinity stress on meat and meat alternatives search behavior, over and above base-rate search behavior. Base-rate search behavior was entered as the first step and (reflected) masculinity stress entered as the second step. Results are summarized first, then presented for the full models in Table 2.

**Table 2:** Study 1: Full regression results for full model with both independent variables added.

	**Unstandardized coefficients**		**Standardized coefficients**		
	**B**	**Std. error**		***t***	**Sig**.
**Dependent variable: Burger**					
Constant	84.15	9.40		8.96	<0.01
Baseline search behavior	−0.58	0.12	−0.39	−4.75	<0.01
Masculinity stress	0.27	0.09	0.24	2.89	0.01
Adjusted *R*^2^ = 0.21, $R_{\text{change}}^{2}$ = 0.06, *F*_(2, 119)_ = 16.823, *p* < 0.01, *F*_change(1, 119)_ = 8.34, *p* = 0.01					
**Dependent variable: BBQ**					
Constant	49.36	10.91		4.53	<0.01
Baseline search behavior	−0.76	0.15	−0.31	−5.07	<0.01
Masculinity stress	0.72	0.11	0.39	6.32	<0.01
Adjusted *R*^2^ = 0.23, $R_{\text{change}}^{2}$ = 0.15, *F*_(2, 206)_ = 31.21, *p* < 0.01, *F*_change(1, 206)_ = 39.98, *p* < 0.01					
**Dependent variable: Steak**					
Constant	32.48	10.70		3.04	<0.01
Baseline search behavior	0.13	0.14	0.09	0.96	0.34
Masculinity stress	0.23	0.11	0.20	2.21	0.03
Adjusted *R*^2^ = 0.03, $R_{\text{change}}^{2}$ = 0.04, *F*_(2, 119)_ = 2.73, *p* = 0.07, *F*_change(1, 119)_ = 4.88, *p* = 0.03					
**Dependent variable: Beyond meat**					
Constant	151.12	12.21		12.37	<0.01
Baseline search behavior	−1.29	0.18	−0.45	−7.29	<0.01
Masculinity stress	−0.79	0.14	−0.35	−5.69	<0.01
Adjusted *R*^2^ = 0.36, $R_{\text{change}}^{2}$ = 0.12, *F*_(2, 173)_ = 49.43, *p* < 0.01, *F*_change(1, 173)_ = 32.35, *p* < 0.01					
**Dependent variable: Impossible burger**					
Constant	−142.45	13.31		10.70	<0.01
Baseline search behavior	−1.20	0.19	−0.42	−6.19	<0.01
Masculinity stress	−0.72	0.16	−0.30	−4.47	<0.01
Adjusted *R*^2^ = 0.30, $R_{\text{change}}^{2}$ = 0.09, *F*_(2, 158)_ = 34.58, *p* < 0.01, *F*_change(1, 158)_ = 19.96, *p* < 0.01					

With “burger” as the dependent variable, the model was significant at the first step [Adjusted *R*^2^ = 0.16, *F*_(1, 120)_ =23.85, *p* < 0.01], and second step [Adjusted *R*^2^ = 0.21, *F*_(2, 119)_ = 16.82, *p* < 0.01]. Importantly, the change in *F* from step one to step two was significant [*F*_change(1, 119)_, *p* = 0.01]. Unstandardized coefficients for the full model show baseline search behavior was negatively associated with “burger” [B = −0.58, SE = 0.12, *p* < 0.01] and masculinity stress was positively associated with “burger” [B = 0.27, SE = 0.09, *p* = 0.01].

With “BBQ” as the dependent variable, the model was significant at the first step [Adjusted *R*^2^ = 0.08, *F*_(1, 207)_ = 18.89, *p* < 0.01], and at the second step [Adjusted *R*^2^ = 0.23, *F*_(2, 206)_ = 31.21, *p* < 0.01]. The change in *F* from step one to step two was significant [*F*_change(1, 206)_ = 39.98, *p* < 0.01]. Unstandardized coefficients for the full model show baseline search behavior was negatively and significantly associated with “BBQ” [B = −0.76, SE = 0.15, *p* < 0.01] and masculinity stress was significantly positively associated with “BBQ” [B = 0.72, SE = 0.11, *p* < 0.01].

With “steak” as the dependent variable, the model was not significant at the first step [Adjusted *R*^2^ = −0.004, *F*_(1, 120)_ = 0.56, *p* = 0.46], or at the second step [Adjusted *R*^2^ = 0.03, *F*_(2, 119)_ = 2.73, *p*=0.07]. The change in *F* from step one to step two was significant [*F*_change(1, 119)_, *p* = 0.03]. Unstandardized coefficients for the full model show baseline search behavior was positively but non-significantly associated with “steak” [B = 0.13, SE = 0.14, *p* = 0.34] and masculinity stress was significantly positively associated with “steak” [B = 0.23, SE = 0.11, *p* = 0.03].

With “Beyond Meat” as the dependent variable, the model was significant at the first step [Adjusted *R*^2^ = 0.24, *F*_(1, 174)_ = 56.36, *p* < 0.01], and second step [Adjusted *R*^2^ = 0.36, *F*_(2, 173)_ = 49.43, *p* < 0.01]. Importantly, the change in *F* from step one to step two was significant [*F*_change_(_1, 173)_, *p* < 0.01]. Unstandardized coefficients for the full model show baseline search behavior was negatively associated with “Beyond Meat” [B = −1.29, SE = 0.18, *p* < 0.01] and masculinity stress was negatively associated with “Beyond Meat” [B = −0.79, SE = 0.14, *p* < 0.01].

With “Impossible Burger” as the dependent variable, the model was significant at the first step [Adjusted *R*^2^ = 0.21, *F*_(1, 159)_ = 43.96, *p* < 0.01], and second step [Adjusted *R*^2^ = 0.30, *F*_(2, 158)_ = 34.58, *p* < 0.01]. Importantly, the change in *F* from step one to step two was significant [*F*_change(1, 158)_, *p* < 0.01]. Unstandardized coefficients for the full model show baseline search behavior was negatively associated with “Impossible Burger” [B = −1.20, SE = 0.19, *p* < 0.01] and masculinity stress was negatively associated with “Impossible Burger” [B = −0.72, SE = 0.16 *p* < 0.01].

As predicted in H1, the potential experience of masculinity stress (as operationalized by actual Google search behavior) was positively associated with the pursuit of red meat like burgers and BBQ, while being negatively correlated with searches for Beyond Meat and the Impossible Burger. We believe this initial evidence lends credence to the theory that men possibly experiencing masculinity stress use food choices, more specifically meat consumption, as a strategic outlet to augment their masculine identity (31). These results also suggest that masculinity stress would preclude the acceptance of meat alternatives which, as discussed, presents a dilemma since choosing them could help men reach their other food-related goals. One limitation of this study is that we did not explicitly measure masculinity stress and used Google search data as a proxy. We reconcile this shortcoming in two additional experiments by measuring masculinity stress to explore how men actually experiencing such discrepancy between their actual and ideal masculinity might, surprisingly, utilize meat alternatives to reconcile this conflict between food-related goals.

### 2.2. Study 2: Gender stress and masculine vs. non-masculine attribute choice within a masculine product

Study 1 finds that, as predicted, masculinity stress was negatively associated with meat alternatives when examining consumer search behavior. In Study 2, we ask whether men who are experiencing a higher degree of masculinity stress might actually be motivated to select meat alternatives when they are able to satisfy their masculinity goal first. To address this question, we ask participants to choose within a masculine product context (a burger) between one with masculine (red meat) and one with non-masculine (non-meat) attributes. In so doing, we explore the possibility that men high on masculinity stress may be *more* inclined to choose a meat alternative when it is presented within a masculine product. We include female consumers as a comparison group, as women do not tend to use meat consumption to make themselves desirable to others (24, 26). We also seek to rule out “new masculinity” beliefs and traditional masculinity as alternative accounts for the proposed effect.

#### 2.2.1. Method

Participants from the United States (*N* = 400) identifying as either male or female and who were not practicing a vegetarian or vegan diet were recruited from Amazon's Mechanical Turk into a continuous (masculinity/femininity stress) by 2 (gender: male vs. female) between-subjects design. Those who identified as currently practicing a vegetarian or vegan diet but slipped through the pre-screen were removed (*N* = 8), as were those who identified as neither male nor female (*N* = 2), resulting in a final sample of 390 [*M*_Age =_ 37.39 (SD = 12.08); 42.2% female].

Participants first completed measures for masculinity stress [or the same measure modified for femininity for female respondents (43); 10 items; 1 = “Strongly disagree” to 7 = “Strongly agree”; α = 0.95 (43)], traditional masculinity [modified for femininity (62); 6 items; 1 = “Not at all masculine/feminine” to 5 = “Very masculine/feminine”; α = 0.84], and the new masculinity inventory [(57); 18 items; α =0.92; 1 = “Strongly disagree” to 7 = “Strongly agree”]. Scale items are presented in Appendix.

Next, in an ostensibly unrelated study, participants were told about a new burger chain, *Big Burger*, that was opening across the country and for which market research was being conducted. They were asked to imagine themselves attending the grand opening, where they could select one of two signature burgers (a real meat burger or a Beyond Meat burger) to consume. The options were described as “a thick and juicy 100% meat [Beyond Meat] patty topped with lettuce, tomato, red onion, and a zesty house-made burger sauce on a fresh-baked bun.” The Beyond Meat option was described as a “plant-based burger,” so respondents knew the choice was a meat alternative. The dependent variable was beef [meat] vs. Beyond Meat [meat alternative] burger choice. Brand and product imagery are presented in Appendix. Respondents then reported their age, were probed for suspicion, and were debriefed.

#### 2.2.2. Results and discussion

**Figure 1:**
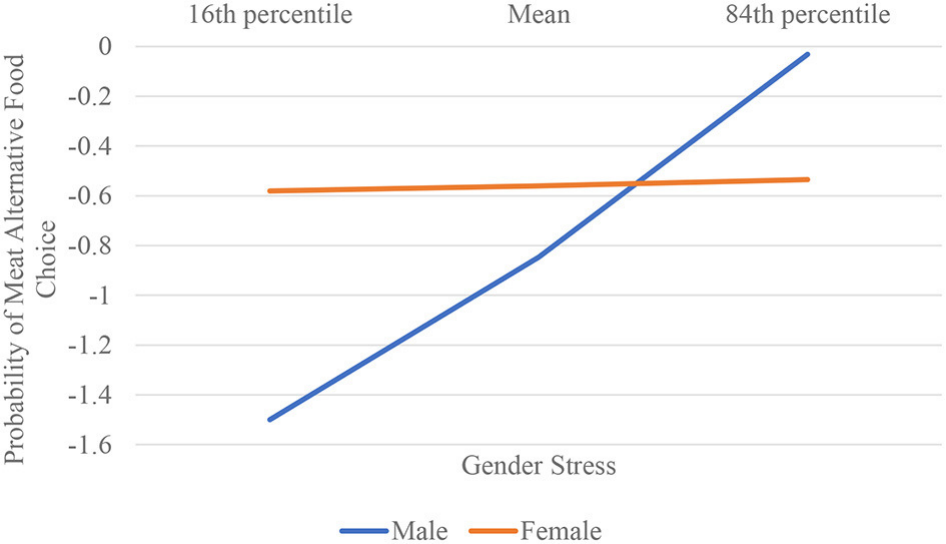
Gender stress by gender on probability of selecting meat alternative within a masculine product (study 2).

Zero-order correlations for all variables are presented in Table 3. We first examined the relationship between masculinity stress (X) and gender (W) on meat alternative choice (Y; 0 = beef burger [meat] and 1 = Beyond Burger [meat alternative]), finding that the overall model was significant (–2LL = 478.61, *p* = 0.0001). Regression coefficients are presented in Table 4. The main effect of masculinity/femininity stress was significant (*Z* = 3.68, *p* = 0.0002; 95% CI: 0.3752–1.231), as was the main effect of gender (*Z* = 2.91, *p* = 0.0036; 95% CI: 0.5324–2.729). Importantly, the interaction between masculinity/femininity stress and gender on choice was significant (*Z* = −2.77, *p* = 0.0056; 95% CI: −0.6748 to −0.1157). Among men, there was a significant relationship between masculinity stress and choice, wherein those higher on masculinity stress were, as theorized, more inclined than those lower on stress to choose a meat alternative when selecting a masculine product (i.e., burger) (*Z* = 4.27, *p* < 0.0001; 95% CI: 0.2208–0.5950). Among females, there was no effect of femininity stress on choice (*Z* = 0.1195, *p* = 0.9048; 95% CI: −0.1951 to 0.2204) (see Figure 1).

**Table 3:** Study 2 descriptives and zero-order intercorrelations.

	**M**	**SD**		**Masc. stress**	**Gender discrep**.	**Discrep. stress**	**Gender**	**Choice**
Masculinity stress	3.45	1.58	Pearson correlation					
			Sig. (2-tailed)					
			N					
Gender discrepancy	3.63	1.69	Pearson correlation	0.938^**^				
			Sig. (2-tailed)	<0.001				
			N	390				
Discrepancy stress	3.27	1.67	Pearson correlation	0.936^**^	0.757^**^			
			Sig. (2-tailed)	<0.001	<0.001			
			N	390	390			
Gender	–	–	Pearson correlation	0.000	0.042	−0.044		
			Sig. (2-tailed)	0.997	0.405	0.388		
			N	390	390	390		
Choice	–	–	Pearson correlation	0.175^**^	0.159^**^	0.169^**^	0.046	
			Sig. (2-tailed)	<0.001	0.002	0.001	0.360	
			N	390	390	390	393	
New masculinity beliefs	5.17	0.97	Pearson correlation	0.112^*^	0.163^**^	0.046	0.105^*^	0.090
			Sig. (2-tailed)	0.028	0.001	0.369	0.038	0.076
			N	390	390	390	391	391

^**^Correlation is significant at the 0.01 level (2-tailed).

^*^Correlation is significant at the 0.05 level (2-tailed).

**Table 4:** Study 2 regression coefficients.

**Regression coefficients (standard errors) analyses (*****N*** = **390)**						
**Dependent variable model [DV** = **choice (red meat** = **0; meat alternative** = **1)]**						
	**Estimate**	**SE**	**Z**	***p***	**LLCI**	**ULCI**
Constant	−3.8652	0.8800	−4.3925	<0.0001	−5.5898	−2.1405
Gender stress	0.8032	0.2184	3.6782	0.0002	0.3752	1.2312
Gender	1.6309	0.5605	2.9099	0.0036	0.5324	2.7294
Gender stress * gender	−0.3953	0.1426	−2.7710	0.0056	−0.6748	−0.1157

Model summary: −2LL = 478.61, Model LL = 20.60, df = 3, p <0.001.

X ^*^ W likelihood ratio of unconditional interaction: X^2^ = 7.79, p = 0.005.

Next, we considered the effect of traditional gender-role beliefs (i.e., masculinity beliefs for males, and femininity beliefs for females) on choice as an alternative account of our findings. However, the overall model was non-significant (*p* = 0.52) and neither the main effects (*p* > 0.30) nor the interaction (*p* = 0.23) were significant. Thus, traditional masculinity/femininity did not substantively influence choice.

We next examined the performance of new masculinity beliefs (57) on choice. It is possible, for example, that beliefs about masculinity which are inconsistent with the hegemonic stereotype may give rise to masculinity stress, and thus masculinity stress may simply be a proxy for new masculinity beliefs. If so, new masculinity beliefs may better explain our theorized effects. Alternatively, there may be a number of factors potentially giving rise to masculinity stress, of which new masculinity beliefs may be one, and masculinity stress may thus perform better at explaining variance in choice behavior.

To assess these possibilities, we employed binary logistic regression among male participants using both masculinity stress and new masculinity as predictor variables, and choice [0 = beef burger (meat) and 1 = Beyond Burger (meat alternative)] as the criterion. Variables were entered using a forward stepwise approach, wherein the best predictor was first pulled into the model (masculinity stress; *B* = 0.41, SE = 0.095, Wald = 18.26, *p* < 0.001). When new masculinity was pulled into the model, masculinity stress remained highly significant (*B* = 0.39, SE = 0.095, Wald = 16.53, *p* < 0.001), and new masculinity accounted for significant variance (*B* = 0.33, SE = 0.165, Wald = 3.97, *p* = 0.046). Thus, while new masculinity beliefs indeed help predict the choice of a beef (vs. Beyond) burger, our findings demonstrate that masculinity stress better predicts this choice than do new masculinity beliefs.

As noted in the intercorrelations table (Table 3), masculinity stress and new masculinity beliefs are mildly, but significantly, correlated (*r* = 0.11, *p* = 0.03), suggesting there may be some, though not extensive, conceptual overlap. This is consistent with our view of masculinity stress as arising from varying factors. As such, we conclude that new masculinity beliefs may give rise to some, but certainly not all, experiences of masculinity stress, and that these constructs should be conceptually distinguished in future research.

### 2.3. Study 3: The mediating effect of masculinity and ethical consumption goals on meat alternative choice within a masculine product

Study 2 finds evidence that, when a product is positioned as masculine, higher masculinity stress is associated with a greater willingness to select a meat alternative product. We propose that this is due to a satisfaction of the masculinity-seeking goal through selection of a “masculine” product, allowing other goal considerations to shape intra-product choices (i.e., meat alternatives); however, Study 2 does not empirically test whether other goals are actually driving the choice of meat alternatives. We address this shortcoming in Study 3 by measuring both masculinity-seeking and ethical consumption goals, predicting that when a product itself is positioned as masculine, manliness goals will not (and ethical consumption goals will) mediate the relationship between masculinity stress and choice of meat alternatives. As a further extension to Studies 1 and 2, we sample male consumers from multiple countries to control for cultural influences on choice.

#### 2.3.1. Method

Male participants (*N* = 500) who were not following any dietary restrictions were recruited from Prolific Academic into a survey design in exchange for payment. Participants who slipped through the pre-screen and identified as non-male (*N* = 4) or who indicated that they followed a non-red meat diet (i.e., vegetarian, vegan or pescatarian; *N* = 11) were removed. Next, respondents who failed one or more bot checks were removed from the sample (*N* = 25). The final sample included 460 men from the United States (77%), and Canada [23%; *M*_Age_ = 31.26 (SD = 10.94)].

Participants were first asked to complete the masculinity stress measure amid a set of filler measures [10 items; 1 = “Strongly disagree” to 7 = “Strongly agree”; α = 0.92 (43)], in an ostensibly unrelated study. Next, participants read about a new restaurant chain, *Macho Burrito*, that would be opening soon, and to imagine themselves attending a grand opening for the restaurant. Specifically, they were told to imagine that at the grand opening they could choose from one of the restaurant's signature “Macho Burritos” filled with either beef or Beyond Meat, described as “*This big burrito comes stuffed full of spiced grass-fed beef [plant-based Beyond Meat], crispy deep-fried onions, peppers, herbs and spices, and our creamy jalapeno Macho Sauce all wrapped in a soft house-made flour tortilla*.” Product imagery is presented in Appendix. The dependent variable was the burrito the respondent chose to sample in the scenario [coded 0 = beef (meat) and 1 = Beyond Meat (meat alternative)]. Once again, Beyond Meat was described as “plant-based,” so respondents knew it was a meat alternative. Finally, respondents were prompted to report on the extent to which masculinity-related goals (“making a high-protein choice,” “making a muscle-building choice,” and “making a strength-building choice”; 3 items; 1 = “Strongly disagree” to 7 = “Strongly agree”; α = 0.89) and ethical consumption considerations (“making an ethical choice” and “making an environmentally conscious choice”; 2 items; 1 = “Strongly disagree” to 7 = “Strongly agree”; α = 0.88) were goals they considered while making their choice, which served as our parallel mediators.

#### 2.3.2. Results and discussion

Zero-order correlations for all variables are presented in Table 5. We tested our hypotheses using PROCESS macro model 4, assessing mediation analysis. Specifically, we examined the effect of masculinity stress (X) on meat alternative choice (Y) through the masculinity-seeking goal (M_1_) and ethical consumption goal (M_2_) in our analysis. Looking first at masculinity-seeking, neither the mediator model examining the effect of masculinity stress on masculinity-seeking goal [*R*^2^ = 0.0034, *F*_(1, 458)_ = 1.58, *p* = 0.21] nor the dependent variable model predicting choice (−2LL = 528.74, df = 2, *p* = 0.50) were significant and, importantly, the confidence interval indicating the presence of an indirect effect included zero (CI: −0.0209 to 0.0071) indicating mediation was not present. Thus, consistent with our theorizing that a masculine product positioning would satisfy masculinity-seeking motives, the extent to which an individual held a masculinity-seeking goal did not mediate the relationship between masculinity stress and meat alternative choice.

**Table 5:** Study 3 descriptives and zero-order intercorrelations.

	**M**	**SD**		**Masc. stress**	**Gender discrep**.	**Discrep. stress**	**Masc. seek**.	**Ethic. Cons**.
Masculinity stress	3.16	1.26	Pearson correlation					
			Sig. (2-tailed)					
			N					
Gender discrepancy	3.37	1.44	Pearson correlation	0.900^**^				
			Sig. (2-tailed)	<0.001				
			N	460				
Discrepancy stress	2.95	1.38	Pearson correlation	0.890^**^	0.603^**^			
			Sig. (2-tailed)	<0.001	<0.001			
			N	460	460			
Masculinity-seeking	3.94	1.60	Pearson correlation	0.059	−0.066	0.177^**^		
Goal			Sig. (2-tailed)	0.210	0.158	<0.001		
			N	460	460	460		
Ethical consumption	3.50	1.66	Pearson correlation	0.110^*^	0.104^*^	0.091	0.364^**^	
Goal			Sig. (2-tailed)	0.019	0.025	0.052	<0.001	
			N	459	459	459	459	
Choice	-	-	Pearson correlation	0.040	0.062	0.009	−0.034	0.464^**^
			Sig. (2-tailed)	0.389	0.185	0.852	0.461	<0.001
			N	460	460	460	460	459

^**^Correlation is significant at the 0.01 level (2-tailed).

^*^Correlation is significant at the 0.05 level (2-tailed).

We next examined the mediating role of ethical consumption goal (M_2_) on the relationship between masculinity stress (X) and meat alternative choice (Y). One participant did not complete the ethical consumption goal items and thus this mediation analysis excludes this individual, leaving 459 participants for the analysis. The mediator model predicting the ethical goal was significant (*R*^2^ = 0.012, *F*_(1, 457)_ = 5.55, *p* = 0.019), as was the dependent variable model predicting choice (−2LL = 425.16, df = 2, *p* < 0.0001). Importantly, in support of our theorizing, the indirect effect of masculinity stress on meat alternative choice was significant (CI: 0.0140 to 0.2083) indicating mediation was present. Higher masculinity stress was associated with a higher ethical goal (*b* = 0.14, *t* = 2.36, *p* = 0.019; CI: 0.0239 to 0.2639), and a higher ethical goal was associated with a greater likelihood of selecting the meat alternative (*b* = 0.73, *Z* = 8.89, *p* < 0.0001; CI: 0.5718 to 0.8954).

## 3. General discussion

This research examines the relationship between masculinity stress and meat alternatives through the lens of goal conflict. We argue that the goal of masculinity among men high on masculinity stress conflicts with other goals like being healthy or making environmentally friendly or ethical food choices. In such situations, men must determine, albeit subconsciously, which goal is salient and, therefore, acted upon at any given point. Our theory suggests that men experiencing masculinity stress will hold the masculinity goal focal while suppressing other goals, thus making the choice of meat alternatives less likely. Using the passive goal guidance model of goal conflict resolution (46), however, we propose that when given the opportunity to signal their masculinity through the selection of a masculine product, these men might be inclined to make a food choice that supports previously inhibited goals, such as choosing meat alternatives. In turn, these new goals shape choice, instead of the masculinity goal.

We present three studies supporting these predictions. In Study 1, we use data from Google Trends to show that search terms that may reflect masculinity stress are positively correlated with search behavior for meat, confirming the findings of previous research. Importantly, we also show that search terms that may reflect masculinity stress are *negatively* correlated with the search for meat *alternatives*, providing additional evidence to recent work [e.g., (35, 55)] suggesting that men concerned with their masculinity should be less likely to pursue meat alternatives. Study 2 draws from the intent of modern forms of meat alternatives to mimic real meat to experimentally assess whether men high on masculinity stress might be inclined to select attributes like meat alternatives that align with competing goals such as ethical consumption, provided they are presented within a masculine product context like a burger or a burrito. Results support this prediction, finding that men high on masculinity stress were *more* likely to choose a meat alternative when selecting a masculine product compared to those low on masculinity stress. Importantly, we additionally show that these results are not apparent for females, and we further rule out new masculinity beliefs as an alternative account for our findings. Finally, Study 3 replicates the focal effect of Study 2 and further tests the prediction that the competing goal of ethical consumption is indeed driving the selection of meat alternatives in the context of masculine products. We present a parallel mediation model with a masculinity-seeking goal and an ethical consumption goal as competing mediators between masculinity stress and food choice. The indirect effect of masculinity stress on meat alternative choice through the masculinity-seeking goal was not significant while the indirect effect through ethical consumption was significant, with higher masculinity stress associated with a greater likelihood of choosing a meat alternative.

### 3.1. Theoretical and practical implications

The current research provides a number of theoretical implications that extend prior work. To begin, our work makes significant contributions to the emerging field of research examining masculinity stress. Masculinity stress is characterized by an enduring worry over one's perceived masculinity (42) and, as an individual difference variable, is not experienced to the same degree amongst all men. While the effects of masculinity stress have been observed across a number of behavioral responses like risky sexual behavior and sexual violence (63) and substance abuse (43), only recently has it been examined within the context of food choice, with Mesler et al. (31) showing its relationship to red meat preference.

Our work extends this research domain by being the first to show, perhaps counterintuitively, that men experiencing masculinity stress also prefer meat *alternatives* under certain conditions. In particular, when men high on masculinity stress also hold goals that conflict with their pursuit of masculinity, they feel compelled to satisfy their masculinity goal first as it is more salient, before switching to alternative goals, like one of ethical consumption that can be achieved by choosing meat alternatives. Thus, the current research is the first to link masculinity stress with goal conflict to provide a new account of what drives preference for meat alternatives—even amongst those who would be least expected to display such preferences.

These results also speak to impression management strategies used by men experiencing masculinity stress. Leary and Kowalski (64) present a two-factor model of impression management consisting of impression motivation and impression construction. Importantly for our work, the motivation to establish an impression is significantly influenced by the goals held by an individual (65), with individuals being more motivated to engage in behaviors that establish a desired impression as goal relevance increases. This suggests that the salience of the masculinity goal held by men experiencing masculinity stress motivates them to engage in behavior designed to signal their masculinity. The subsequent stage of impression construction is accomplished when the person acts in a manner consistent with this goal, such as through the selection of a masculine product, which allows them to establish their desired image (66). Thus, our work suggests that men high on masculinity stress use their selection of masculine products as the construction of a desired masculine image. In other words, “you are what you eat” (67), and men experiencing distress about their masculinity are more likely to choose a (manly) product reflective of their preferred self.

Such self-presentation techniques can, however, have detrimental health and wellbeing consequences (68). This is especially true with the consumption of red meat (19), which has been linked to increased risks of cancer, obesity, and cardiovascular disease (1, 3). Though we note again that the objective health benefits of PBMAs are held in question (9, 13), we believe our work, by showing how men can still signal their masculinity through the selection of a masculine product, is a step in the right direction for how they may do so with products that have potentially healthier attributes than red meat, and definitively fewer ethical and environmental concerns.

It important to note that recent research (26) suggests that men who wish to signal their masculinity might actually be more likely to do so through ethical/sustainable consumption, such as choosing “green” products that signal a higher status, which might translate to the selection of sustainable food choices like meat alternatives. This possible link between ethical consumption goals and masculinity goals is supported by the correlation between these two measures in Study 3 (0.364; *p* < 0.01; see Table 5), suggesting that the men in our study might have simultaneously held these two goals as we suggest. It could thus be potentially argued that consuming meat alternatives to obtain status is consistent with the desire to display one's masculinity, which would mean that there is no conflict between masculinity and meat alternative selection. However, the existing research presented suggests that meat alternatives are viewed as less masculine and, as such, the goals of masculinity-signaling and ethical food choices are often (though not necessarily always) in conflict with one another. We note that the products examined herein are relatively new food innovations, and that cultural views of these products may change as uptake diffuses throughout the market. Thus, more work needs to be done to validate this possible connection and to determine if men (particularly those experiencing masculinity stress) view modern forms of meat alternatives as masculinity-signaling sustainable food products, and whether such an effect will change over time.

These findings distinguishing between the effects of masculinity stress at the product (i.e., burger/burrito) and attribute (i.e., red meat/meat alternatives) levels are also an important contribution of our work. We show that, when considering their competing goals like being masculine and eating ethically, men are making distinctions on how they might be able to accomplish both. One way that our findings illustrate this might be possible is through prioritizing one (the product) over the other (attribute), which dictates initial behavior. Outside of the food domain, this could manifest in products such as Ford's F-150 Lightning, which is an electric vehicle that gives the consumer the opportunity to select a masculine product like a pickup truck, while offering the flexibility to meet other goals like reducing carbon emissions. While we do not test whether this consideration is explicit or implicit, the research on goal conflict suggests that this occurs at a subconscious level, with men not necessarily aware of how the process operates. Future research should continue this examination to determine whether men are aware of the trade-offs being made when they select a masculine product with potentially fewer masculine attributes.

Practically speaking, our results also provide guidance and direction on how manufacturers might promote meat alternatives so as to encourage acceptance among men experiencing masculinity stress. Manufacturers of these products might be well-served by promoting their products as masculine alternatives to real meat. That is, the products can be positioned as giving men the best of both worlds, allowing them to appear masculine and achieve their competing goals. Industry stalwarts Beyond Meat and Impossible Foods have already begun this messaging, signing such masculinity-signaling celebrity endorsers as Kevin Hart, Chris Paul, Snoop Dogg, Kim Kardashian, and professional wrestler Nikki Bella (69).

### 3.2. Limitations and future directions

While there are numerous strengths in this research, such as using both real consumer search behavior in addition to experimental data, we acknowledge there were also limitations that future research should consider. One potential future direction is considering whether consumption is done in public or private. Namely, how is the goal satisfied—through the explicitly external impression management motivation or an internal process such as goal satisfaction? We argue herein that these two are inextricably linked, such that the impression management motivation leads to behavior that satisfies one's masculinity goal, which then frees them to pursue other goals. In the current research, however, we do not assess the impression management motivation, which future work should endeavor to do utilizing previous work on how goal progress and satisfaction impacts future behavior [i.e., (70)].

Building on this, researchers could also consider whether the attribute within the product (e.g., being plant-based) is visible or non-visible. For example, if a plant-based burger looks exactly like a real-meat burger, is the masculine goal still satisfied? Preliminary results from our research suggest this may be the case, however, further research should assess this in other products while considering features such as color, scent, and other focal attributes which might influence perception and choice. Future research may also investigate how making other, typically feminine, products, services, or behaviors more masculine impacts the acceptance among men experiencing masculinity stress. For example, could skin care routines, driving smaller vehicles, or talking about emotions be presented as masculine to both satisfy masculinity goals and potentially improve men's wellbeing? While our results do not speak to the likelihood of this phenomenon, such research would be important in further elucidating the effects of masculinity stress, and how men experiencing negative perceptions of their manhood might be encouraged to engage in less harmful behavior.

### 3.2. Conclusions

The rise of modern forms of meat alternatives in recent years suggest that they will be here for a long time to come. Our work presents a novel perspective on the acceptance of these products among men who might be least expected to do so—men who believe they are insufficiently masculine. This is particularly important given the noted health impacts of consuming too much red meat. The studies herein highlight the importance of understanding the role of masculinity stress and goal conflict in this process, while offering directions for future researchers to further examine and extend this work.

## Data availability statement

The raw data supporting the conclusions of this article will be made available by the authors, without undue reservation.

## Ethics statement

The studies involving human participants were reviewed and approved by University of Nevada, Reno Institutional Review Board. The patients/participants provided their written informed consent to participate in this study.

## Author contributions

All authors listed have made a substantial, direct, and intellectual contribution to the work and approved it for publication.

## Footnotes

^1^We use the terms plant-based meat alternatives, PBMAs, and meat alternatives interchangeably in this paper.
