# Supplementary material for: This meat or that alternative? How masculinity stress influences food choice when goals are conflicted
Source: Front Nutr. 2023 Mar 7;10:1111681. doi: 10.3389/fnut.2023.1111681 (PMC10028087; doi:10.3389/fnut.2023.1111681)

**Appendix**

| **Scale** | **Items** | |
| --- | --- | --- |
| **Masculinity Stress (Reidy et al., 2016)** | | |
|  | 1. I am less masculine than the average guy. | |
|  | 2. Compared to my guy friends, I am not very masculine. | |
|  | 3. I wish I was more “manly.” | |
|  | 4. Most women I know would say that I am not as masculine as my friends. | |
|  | 5. I wish I was interested in things that other guys find interesting. | |
|  | 6. Most women would consider me to be less masculine than the typical guy. | |
|  | 7. I worry that people judge me because I am not like the typical man. | |
|  | 8. Most guys would think I am not very masculine compared to them. | |
|  | 9. Sometimes I worry about my masculinity. | |
|  | 10. I worry that women find me less attractive because I’m not as macho as other guys. | |
| **Traditional Masculinity/Femininity (Kachel et al., 2016)** | | |
|  | 1. I consider myself as… | |
|  | 2. Ideally, I would like to be… | |
|  | 3. Traditionally, my interests would be considered as… | |
|  | 4. Traditionally, my attitudes and beliefs would be considered as… | |
|  | 5. Traditionally, my behavior would be considered as… | |
|  | 6. Traditionally, my outer appearance would be considered as… | |
| **New Masculinity Inventory (Kaplan et al., 2017)** | | |
|  | 1. Men should make an effort to each nutritious foods because they are paying attention to their bodies, and not only for health reasons. | |
|  | 2. Men should try to achieve full harmony between mind and body. | |
|  | 3. A man should come to know himself through paying attention to his body and its needs. | |
|  | 4. Men should emphasize dialogue and listening to others as a way of life. | |
|  | 5. Society’s definition of masculinity is partial and too restrictive. | |
|  | 6. Men should follow their hearts and inclinations, even in ways that society deems “unmanly.” | |
|  | 7. I appreciate men who are willing to take up ”feminine” or less profitable jobs in order to increase their personal satisfaction. | |
|  | 8. Men should enjoy their sexual experiences, regardless of how they “perform” sexually. | |
|  | 9. The distinction between masculine and feminine characteristics and roles is damaging for both men and women. | |
|  | 10. Men should let themselves experience a variety of sexual behaviours, even ones that are not in line with the sexual roles expected of men. | |
|  | 11. Men should be able to express their feelings at work the same way they do at home or with friends. | |
|  | 12. Men should let themselves express the various aspects of their personality with equal ease at work, at home, and with friends. | |
|  | 13. Men should be encouraged to share their feelings and concerns more often and more openly with others. | |
|  | 14. Men should constantly search for meaning and strive for personal development and growth. | |
|  | 15. Involvement in hands-on childcare should play a significant role in men’s self-realisation as fathers. | |
|  | 16. Helping one’s children develop their true selves is a more important part of fatherhood than focusing on their financial well-being. | |
|  | 17. A man’s career should not come at the expense of his family, friends, and hobbies. | |
|  | 18. A man should be able to give priority to the career of his spouse, even if his own career slows down as a result. | |
| **Ethical and Sustainable Food Goals (developed for this study)** | | |
| Consider the food choice you made. In making that choice, how important were the following goals to you? | | |
|  | | 1. Making an ethical choice.  2. Making an environmentally conscious choice.  3. Making a muscle-building choice.  4. Making a high-protein choice.  5. Making a performance-enhancing choice. |

Study 2 Brand and Product Imagery


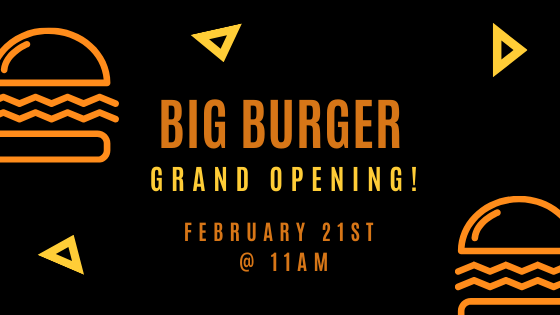

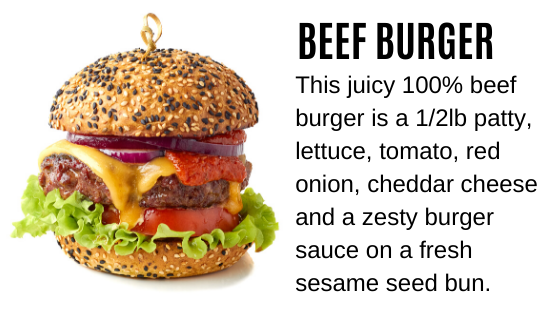

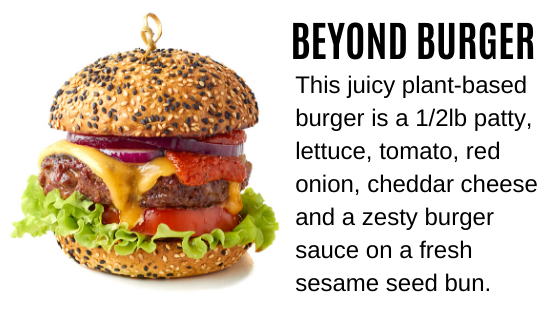


Study 3 Product Imagery


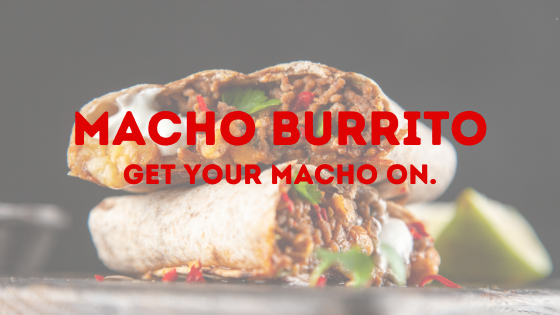

Supplement: Supplementary file 1 [file Data_Sheet_1.docx]
